# Polymer Nanoparticles with 2-HP-β-Cyclodextrin for Enhanced Retention of Uptake into HCE-T Cells

**DOI:** 10.3390/molecules29030658

**Published:** 2024-01-31

**Authors:** Zhenmiao Qin, Baohua Li, Qiyi Deng, Yifeng Wen, Shiquan Feng, Chengcheng Duan, Beicheng Zhao, Hailong Li, Yanan Gao, Junfeng Ban

**Affiliations:** 1 Hainan Key Laboratory for Research and Development of Tropical Herbs, School of Pharmacy, Hainan Medical University, Haikou 571199, China; qzm2020@hainmc.edu.cn (Z.Q.); 17330925753@163.com (S.F.); 19171632173@163.com (C.D.); zbc19846793427@163.com (B.Z.); lihailong@hainmc.edu.cn (H.L.); 2 Guangdong Provincial Key Laboratory of Advanced Drug Delivery Systems, Guangdong Pharmaceutical University, Guangzhou 510006, China; bbaohua66@163.com (B.L.); 13650929045@163.com (Q.D.); 13570252336@163.com (Y.W.)

**Keywords:** ocular delivery systems, transcellular barrier mechanism, corneal permeability, ocular bioavailability

## Abstract

Triamcinolone acetonide (TA), a medium-potency synthetic glucocorticoid, is primarily employed to treat posterior ocular diseases using vitreous injection. This study aimed to design novel ocular nanoformulation drug delivery systems using PLGA carriers to overcome the ocular drug delivery barrier and facilitate effective delivery into the ocular tissues after topical administration. The surface of the PLGA nanodelivery system was made hydrophilic (2-HP-β-CD) through an emulsified solvent volatilization method, followed by system characterization. The mechanism of cellular uptake across the corneal epithelial cell barrier used rhodamine B (Rh-B) to prepare fluorescent probes for delivery systems. The triamcinolone acetonide (TA)-loaded nanodelivery system was validated by in vitro release behavior, isolated corneal permeability, and in vivo atrial hydrodynamics. The results indicated that the fluorescent probes, viz., the Rh-B-(2-HP-β-CD)/PLGA NPs and the drug-loaded TA-(2-HP-β-CD)/PLGA NPs, were within 200 nm in size. Moreover, the system was homogeneous and stable. The in vitro transport mechanism across the epithelial barrier showed that the uptake of nanoparticles was time-dependent and that NPs were actively transported across the epithelial barrier. The in vitro release behavior of the TA-loaded nanodelivery systems revealed that (2-HP-β-CD)/PLGA nanoparticles could prolong the drug release time to up to three times longer than the suspensions. The isolated corneal permeability demonstrated that TA-(2-HP-β-CD)/PLGA NPs could extend the precorneal retention time and boost corneal permeability. Thus, they increased the cumulative release per unit area 7.99-fold at 8 h compared to the suspension. The pharmacokinetics within the aqueous humor showed that (2-HP-β-CD)/PLGA nanoparticles could elevate the bioavailability of the drug, and its *C_max_* was 51.91 times higher than that of the triamcinolone acetonide aqueous solution. Therefore, (2-HP-β-CD)/PLGA NPs can potentially elevate transmembrane uptake, promote corneal permeability, and improve the bioavailability of drugs inside the aqueous humor. This study provides a foundation for future research on transocular barrier nanoformulations for non-invasive drug delivery.

## 1. Introduction

Triamcinolone acetonide (TA) is a glucocorticoid and is commonly used to treat various ophthalmic conditions, including macular edema, age-related macular degeneration, and proliferative vitreoretinopathy by intravitreally injecting a trimethoprim suspension. However, TA is easily susceptible to retinal detachment, retinal hemorrhage, endophthalmitis, and other adverse effects induced by high-dose administration. Consequently, identifying a suitable drug delivery system is critical to addressing this problem. Ophthalmic preparations for topical administration are delivered to intraocular tissues through the corneal, conjunctival, and uveo-scleral routes [1,2]. The cornea is the most significant static barrier in the front of the eye. It is divided into epithelial, anterior elastic, stromal, posterior elastic, and endothelial cell layers, of which the corneal epithelial and stromal layers are the main barriers to drug delivery [3,4]. The corneal epithelium comprises 3–6 tightly connected layers of lipid-rich epithelial cells behaving as a barrier against most drugs, thereby retaining most drugs in the epithelium [2,5,6]. Therefore, prolonging the retention time of the drug in the cornea to reduce the loss of the drug due to tear flushing, thus improving the ocular surface, can enhance the permeability of the drug in the ocular tissue for effective delivery to the target site [7,8]. The biocompatible and biodegradable polymeric material PLGA has been approved by the FDA for ocular drug delivery systems and is the most commonly used among nanocarrier systems [9]. Cell-penetrating peptides (CPPs), usually short peptides with high permeability in biological membranes, could facilitate the intracellular delivery of hydrophilic proteins and nucleic acids. Jiang et al. [10] designed a flexible octopus-like octavalent penetrating protein (8VP). It had a multi-armed PEG core and penetrating binding proteins at both ends of the PEG arm as extension tentacles for facilitating the concentration and delivery of therapeutic nucleic acids. The 8VP stably compressed siRNA into polymorphs less than 100 nm in size and positively charged, enhancing cellular uptake efficiency by nearly 100% and transfection rates by more than 75%. When 8VP was injected inside the conjunctival capsule, it rapidly distributed siRNA within the retina through a non-corneal pathway, and the retention time became longer than six hours. In a retinoblastoma tumor-bearing mouse model, a topical drip of 8VP/siRNA effectively inhibited intraocular tumor protein expression and was tolerated well inside the eyes. Additionally, it has been reported that the nanotopical eye drop delivery system is influenced by various factors, including pH [11], particle size [12,13], electric charge [14], and surface modification [15,16], resulting in differences in drug absorption.

Nanoparticle preparations crossing the ocular barrier with PLGA carriers are susceptible to tear elimination. Therefore, modifying the surface properties of PLGA nanoparticles is essential. Cyclodextrins, a versatile, functional excipient, have an external hydroxyl group forming hydrogen bonds between molecules and self-assemble to develop aggregates in aqueous solutions. The enhanced membrane permeation mechanism of cyclodextrins is associated with their improved electrostatic adsorption and van der Waals forces to augment drug availability for biofilm surface adsorption. This leads to an effective drug permeability enhancement [17]. It was demonstrated that preparing cyclodextrin–PLGA aggregates could be one of the strategies to improve the bioavailability of drugs for topical administration on the ocular surface. For drug–cyclodextrin inclusions, the hydrophobic drug as a guest molecule can drive the aggregates, forming nano- and micron-sized aggregates. In a previous study, a PLGA vehicle was used to prepare triamcinolone acetonide–cyclodextrin complex nanoparticles. The peak concentration *C*_max_ and AUC_0–6h_ in the aqueous humor were, respectively, 36.8 and 44.9 times higher than those in the triamcinolone acetonide suspension group after ocular administration within rabbits [18]. The preparation of cyclodextrin–PLGA aggregates improves the bioavailability of drugs for topical administration on the ocular surface, easing the self-assembly of drug–cyclodextrin inclusion complexes in an aqueous solution to form aggregates [19]. 

However, the in vivo behavior of nanoformulations is based on data obtained by monitoring their pharmacokinetics and tissue distribution. These data cannot entirely elucidate the specific processes of in vivo nanodelivery systems. Moreover, the scientific questions regarding the internalization or in vivo transport of nanodelivery systems in their complete form and the specific transmembrane transport mechanisms of nanodelivery systems have not been specifically answered. Since the absence of a precise mechanism blurs drug development, the transmembrane transport mechanisms of nanodelivery systems need to be elucidated to pave the way for rational drug design. The adhesion of cyclodextrin-coated nanoparticles has been represented in previous studies. Moreover, it also facilitates drug absorption by improving retention time on the ocular surface. In this study, PLGA nanoparticles were the subject, and rhodamine-B (Rh-B) was the model drug used to prepare a nanodelivery system for investigating their uptake and transport behavior. In addition, the mechanism of nanodelivery uptake due to cellular uptake, transmembrane transport, intraocular delivery, and local uptake was also demonstrated, thereby validating the quantitative changes in biological effects.

## 2. Results and Discussion

### 2.1. 2-HP-β-CD/PLGA Nanoparticle Characterization and Evaluation

**Figure 1:**
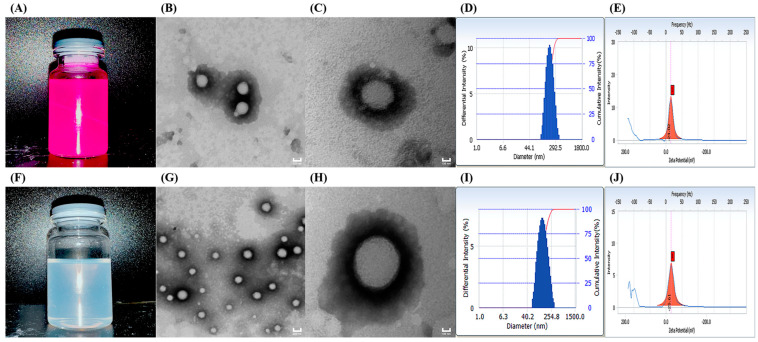
Appearance (**A**,**F**); transmission electron microscopy ((**B**,**G**) 2 × 10^4^, (**C**,**H**) 5 × 10^4^); size distribution (**D**,**I**); and zeta potential (**E**,**J**) results of Rh-B-(2-HP-β-CD)/PLGA NPs (**A**–**E**) and TA-(2-HP-β-CD)/PLGA NPs (**F**–**J**).

The particle size and potential of NPs are the predominant elements affecting their cellular transport and corneal permeation. When the particle size is below 200 nm, it is easier to reach the atrial fluid through the cornea [20]. As shown in Figure 1A,F, Rh-B-(2-HP-β-CD)/PLGA NPs produced a purple emulsion, and TA-(2-HP-β-CD)/PLGA NPs presented a blue opalescent appearance. Figure 1D,I demonstrate that the nanoparticle eye drops prepared using different model drugs possessed a uniform particle size and narrow distribution range, with homogeneous and stable systems. The measurement results of the three samples (each measured three times in parallel) are represented in Table 1. These indicated the low median diameter fluctuation of the nanosystem, with a PDI of around 0.1 and stable zeta potential values. TEM magnifications of 2 × 10^4^ (Figure 1B,G) and 5 × 10^4^ (Figure 1C,H) depicted the spherical, uniform, and dispersed morphology of the NPs. The particle size observed at a 5 × 10^4^ magnification was more significant than the Delas Nano C laser particle size determination. This could be associated with the desiccation of the sample during pre-treatment for the TEM measurement. The optimum range for ophthalmic preparations is 40–50 mN·m^−1^ [21]. The PLGA NPs modified with 2-HP-β-CD enhanced the surface tension from 40.58 ± 1.53 mN·m^−1^ to 42.9 ± 1.0 mN·m^−1^, compliant with the requirements. 2-HP-β-CD oligosaccharide was incorporated to enable significant contact and sufficient distribution at the corneal site after administering eye drops. HPLC revealed that the PLGA nanoparticles modified with 2-HP-β-CD had a higher entrapment efficiency (73.73% ± 4.8%) and drug loading capacity (7.39% ± 0.48%).

### 2.2. In Vitro Drug Release Study

**Figure 2:**
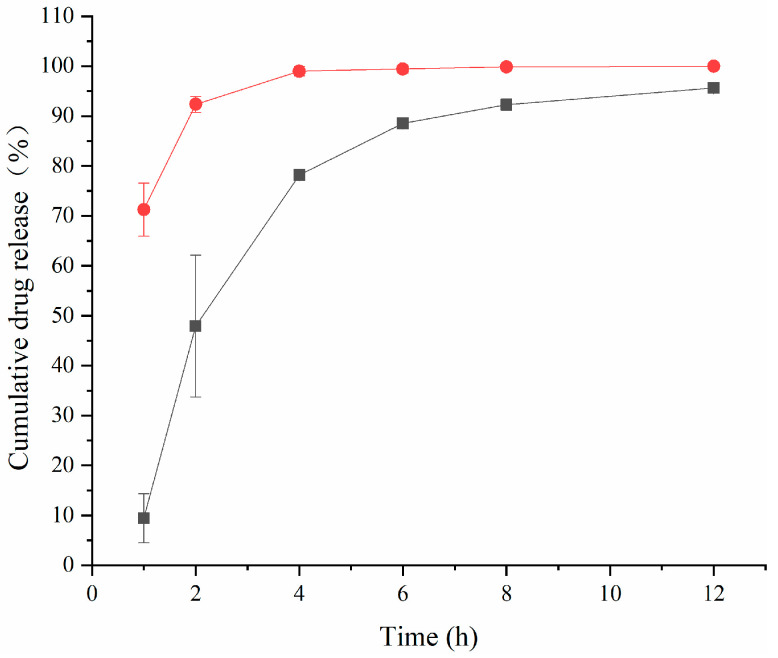
The cumulative release rate of triamcinolone acetonide in artificial aqueous humor (mean ± SD, *n* = 6) (●: TA aqueous suspension; ■: (2-HP-β-CD)/PLGA-loaded nanoparticles).

Figure 2 shows that the cumulative release rate of the TA aqueous suspension was as high as 92.34% ± 1.61% at 2 h. However, TA-(2-HP-β-CD)/PLGA NPs could reach 88.53% ± 0.72% in 6 h. Two phases characterized the release pattern: firstly, during the first 4 h, there was an abrupt release phase, mainly due to the partial adsorption of the pharmaceuticals on the nanoparticle surface. Moreover, there was rapid distribution from the polymer to the aqueous phase when there was contact with the release medium. Then, it entered into a slow-release phase, which was maintained for a more extended time period. The main reason for the release of drugs was that the polymer matrix came into sufficient contact with the release medium and became swollen and loosened, leading to the diffusive release of the encapsulated medicines. The first phase of the NPs (the abrupt release phase) facilitated the delivery of effective therapeutic concentrations in a short time period. In contrast, the second phase (the slow-release phase) maintained the effective drug concentrations for a certain duration.

In order to visualize the mechanism and pattern of drug release from the nanoparticles, the release data could be fitted to four commonly used mathematical models, including zero-order, first-order, Higuchi, and Ritger–Peppas kinetics emission models. The model fits for each formulation are represented in Table 2. The TA aqueous suspension and the TA-(2-HP-β-CD)/PLGA NPs were similar according to the first-order kinetic release model in vitro. This coincides with the occurrence of a “burst effect” in the first half of the release curve described above, which promoted the achievement of effective therapeutic concentrations in a short period of time.

### 2.3. Permeability Evaluation of Isolated Corneas 

**Figure 3:**
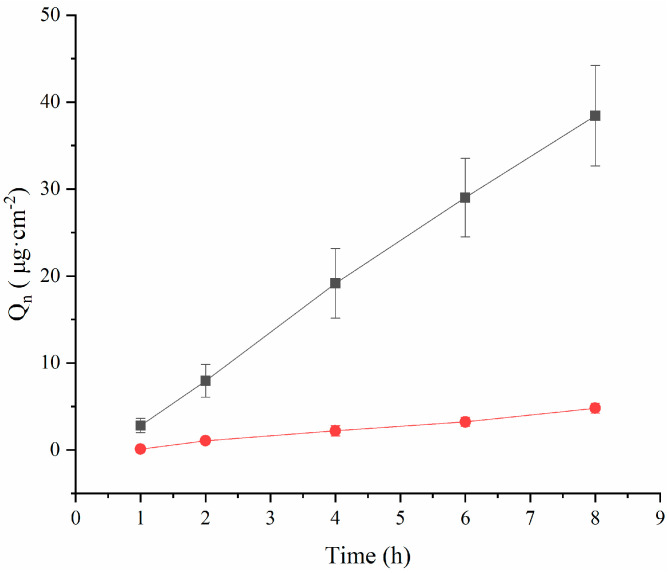
The permeability of triamcinolone acetonide across excised rabbit corneas (mean ± SD, *n* = 3). (●: TA aqueous suspension; ■: (2-HP-β-CD)/PLGA-loaded nanoparticles).

The results of the ex vivo corneal permeation are demonstrated in Figure 3, where the osmotic accumulation of the drug increased consistently in a linear relationship with time. Based on cumulative permeation, the TA-(2-HP-β-CD)/PLGA NP eyedrops demonstrated an accumulation per unit area of 38.44 ± 5.77 μg·cm^−2^ at 8 h. This accumulation was significantly higher (*p* < 0.01) than that of the aqueous tretinoin control (4.81 ± 0.58 μg·cm^−2^). The corneal permeability of lipophilic drugs is stronger than that of hydrophilic drugs [8]. However, lipophilic drugs have limited use in ocular drug delivery due to their poor solubility and easy clearance by the RES system. Due to having many hydroxyl groups, cyclodextrins can improve the solubility of insoluble drugs without affecting their lipophilicity and enhance the ability of the carrier to penetrate biological membranes. Simultaneously, the cyclodextrin surface is hydrophilic and can carry lipophilic carriers or drugs through the tear film layer, thereby increasing their retention time in front of the cornea. Therefore, the drug can be distributed from the tear film layer to the corneal surface, enhancing its concentration on the surface of the eye wall and improving its corneal permeability.

The accumulated permeation per unit area (*Q*) was fitted through the formula with time (t) to calculate the value of *P_app_* and *J_ss_* (Table 3), showing that the P_app_ of the TA aqueous solution was (8.35 ± 1.02) × 10^7^ cm·s^−1^ at 8 h. This outcome was due to the inherent hydrophobic nature of the drug limiting its diffusion through the cornea. However, the *P*_app_ value of the TA-(2-HP-β-CD)/PLGA NPs was 8.24-fold higher than that of the TA aqueous solution. Moreover, the *J_ss_* value of the TA-(2-HP-β-CD)/PLGA NPs was 8.31-fold higher than that of the TA aqueous solution (above *p* < 0.01). Meanwhile, the TA-(2-HP-β-CD)/PLGA NPs were 7.33-times more effective than the TA aqueous solution in determining corneal retention.

Therefore, the 2-HP-β-CD-modified nanoparticles were functional in promoting drug penetration by forming a drug reservoir within the corneal epithelium and maintaining a saturated drug concentration in the aqueous layer of the tear fluid. They also regulated the driving force for drug diffusion within the atrial fluid.

### 2.4. Pharmacokinetic Study of In Vivo Aqueous Humor

The pharmacokinetic parameters in aqueous humor are shown in Table 4. The AUC of the TA-(2-HP-β-CD)/PLGA NPs was 52.53-fold, the MRT 1.03-fold, and the VRT 2.07-fold higher than those of the TA aqueous suspension within the aqueous humor. The 2-HP-β-CD-modified PLGA NPs were competent in developing pharmaceutical depositories in front of the cornea. They were impervious to tear washout, permitting the nanoparticles to retain a specific drug concentration and sustained release in the aqueous humor. This could be explained by the hydrophilic nature of 2-HP-β-CD and its ability to carry insoluble lipophilic molecules to penetrate the aqueous layer of the tear film. Therefore, the PLGA nanoparticles modified with 2-HP-β-CD could augment the drug concentration on the surface of the ocular wall, strengthen its entry into the eye, and promote bioavailability.

**Figure 4:**
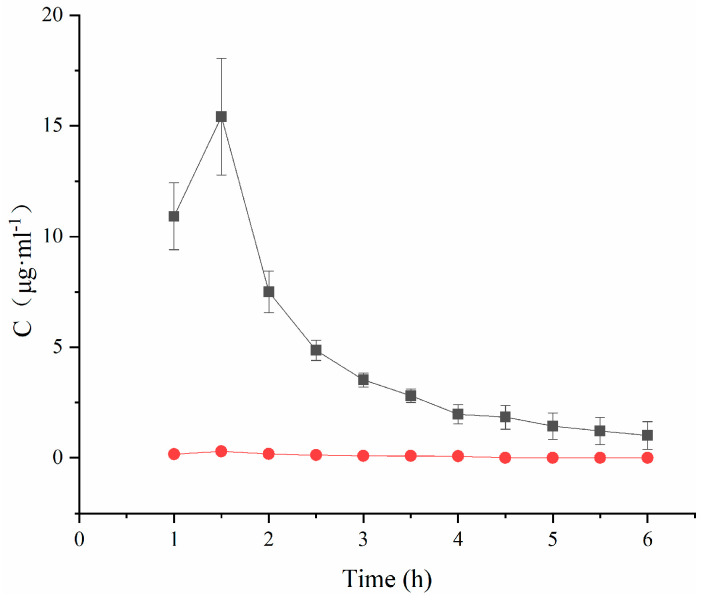
Concentration–time curves of triamcinolone acetonide in aqueous humor (mean ± SD, *n* = 3). (●: TA aqueous suspension; ■: (2-HP-β-CD)/PLGA-loaded nanoparticles).

Plotting the TA concentration and time to obtain Figure 4 and analyzing the data revealed that the peak concentration C_max_ in the TA-(2-HP-β-CD)/PLGA NP group was 51.91 times higher than that in the aqueous solution group. This meant that the nanoparticles benefited from the absorption of the drug, especially after modification with 2-HP-β-CD. The nanoparticles remarkably elevated the concentration of drugs in the atrial water (*p* < 0.01) and increased the local bioavailability.

### 2.5. Transcellular Barrier Uptake Studies with 2-HP-β-CD/PLGA Complex Nanoparticles

**Figure 5:**
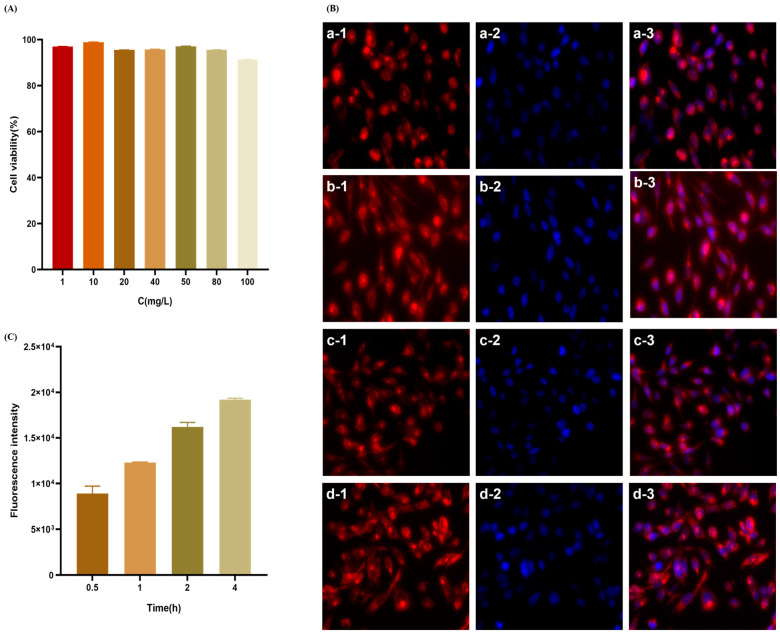
In vitro cytotoxicity of (2-HP-β-CD)/PLGA NPs against HCE-T cells (**A**) and the uptake of (2-HP-β-CD)/PLGA NPs at different time points by HCE-T cells were observed by an inverted fluorescence microscope. (**B**) The red fluorescence signal indicates rhodamine B-labeled nanoparticles, and the blue fluorescence signal shows hoechst33342-labeled nuclei (a: 0.5 h; b: 1 h; c: 2 h; d: 4 h). (**C**) Nanoparticle uptake at different time points by HCE-T cells was measured through flow cytometry (*n* = 3).

The cell survival rate was evaluated using the CCK8 method at concentrations ranging from 1 to 100 μg·mL^−1^ to investigate the transport of the nanodelivery system across the ocular barrier. The results are depicted in Figure 5A. The survival rate of HCE-T cells in (2-HP-β-CD)/PLGA nanoparticles exceeded 90%, establishing that the nanodelivery system was non-toxic to HCE-T cells at this concentration range.

It has been reported that nanoparticle uptake by cells begins with adhesion to the cell membrane, followed by endocytosis through the surface of the cell membrane and then into the cell. The rate and mechanism of uptake are influenced by the particle size, potential value, and surface properties of the nanoparticles. The uptake behavior of the nanoparticle delivery system was studied based on the construction of an HCE-T epithelial cell barrier model. The analysis of Figure 5B revealed that the uptake of NPs into the cells was primarily distributed in the cytoplasm and was kept out of the nucleus. HCE-T cells exhibited a moderate uptake of (2-HP-β-CD)/PLGA NPs at 0.5 h. The fluorescent signal in the HCE-T cells increased continuously with increasing ingestion time. The uptake of NPs was time-dependent, increasing with time over 4 h. Flow cytometry facilitates the efficient, accurate, and multi-parameter quantitative analysis of cells in rapid linear motion. Depending on the X-mean values of FCM (Figure 5C), the ingestion of (2-HP-β-CD)/PLGA NPs by HCE-T cells was enhanced continuously with the intake duration over 4 h. These results were consistent with those observed by IXM. 2-HP-β-CD can increase drug penetration through biological membranes by disrupting the membrane structure or complexing with lipophilic components present in the cell membrane to enhance the corneal permeability of the drug [22,23].

**Figure 6:**
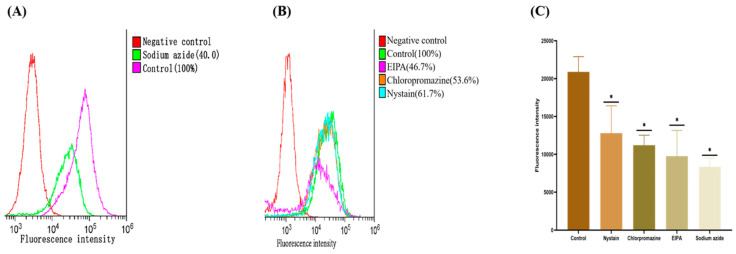
The result of cellular uptake: (**A**) the effects of inhibitors of active transport pathways on uptake of nanoparticles by HCE-T cells; (**B**) the effects of inhibitors of different endocytic transport pathways on uptake of nanoparticles by HCE-T cells; (**C**) difference in the uptake of (2-HP-β-CD)/PLGA NPs by HCE-T cells. * indicates significant differences between the experimental and the control groups (*n* = 3, *p* < 0.01).

We evaluated the effectiveness of different inhibitors of the mechanism of nanoparticle uptake to investigate the mode of nanoparticle uptake. The effect of active transport on nanoparticle uptake was evaluated using sodium azide, an active transport inhibitor. The results indicated (Figure 6A,C) that the uptake of (2-HP-β-CD)/PLGA NPs reduced by 60.0%, becoming significantly lower than that of the normal group (*p* < 0.01). The intake of (2-HP-β-CD)/PLGA NPs was more dependent on the active transport mode, further demonstrating that the (2-HP-β-CD)/PLGA NPs combined with mucin on the HCE-T cells, thereby facilitating the transport of drugs through the cornea. Endocytosis transport processes mediated by the pinocytosis pathway could be triggered when the size of nanoparticles is over 150 nm [24]. It was indicated (Figure 6B,C) that (2-HP-β-CD)/PLGA NPs were primarily absorbed through macropinocytosis. At the same time, cellular ingestion was partially mediated by clathrin-mediated endocytosis and caveolin/lipid raft structure endocytosis. 5-(N-ethyl-N-isopropyl) amiloride (EIPA) effectively inhibited macropinocytosis-induced endocytosis. Its incorporation resulted in the HCE-T cell uptake of (2-HP-β-CD)/PLGA NPs being significantly (*p* < 0.01) inhibited by 53.3%, demonstrating that the uptake of (2-HP-β-CD)/PLGA NPs was dominated by macropinocytosis. Chlorpromazine potentially prevents the integration of clathrin into cell membranes, preventing the formation of clathrin-structure-mediated endocytosis so that the endocytosis of macromolecules into the cell becomes difficult [25]. The data indicated that the HCE-T cell feeding of (2-HP-β-CD)/PLGA NPs was significantly inhibited by 46.4% (*p* < 0.01) through the addition of Chlorpromazine, implying that clathrin-structure-mediated endocytosis also played a significant role in the entry of NPs into the cells. Nystain is a cholesterol chelator reducing the caveolin/lipid raft structure quantity on the cell membrane surface. This helps block caveolin/lipid raft-structure-mediated endocytic transport [26,27]. The absorption of (2-HP-β-CD)/PLGA NPs into HCE-T cells was significantly reduced by 38.3% (*p* < 0.01) after adding Nystain. This supports the occurrence of nanoparticle-dependent endocytosis on the caveolin/lipid raft structure. The endocytosis mediation by macropinocytosis, clathrin, and the caveolin/lipid raft structure are all energy-depleting delivery procedures, viz., active transport modes. Moreover, multiple pathways regulated the NP entry into the cells, and 2-HP-β-CD-modified PLGA nanoparticles showed enhanced uptake into cells and an altered mode of entering cells.

## 3. Materials and Methods

### 3.1. Materials

Triamcinolone acetonide (TA, 99.9%) was obtained from Hehui Pharmaceutical Group Co., Ltd. (Tianjin, China). Rhodamine B (Rh-B) was obtained from Damao Chemical Reagent Factory (Tianjin, China); Polyvinyl alcohol (PVA) from Kuraray International Trading Co., Ltd. (Shanghai, China); 2-Hydroxypropyl-β-cyclodextrin (2-HP-β-CD, substitution degree 4.96) from Qianhui Biotechnology Co., Ltd. (Zibo, China); Poly(lactic acid glycolic acid) copolymer (PLGA, 50:50, M_w_ 10,000 Da) from JFK Biotechnology Co., Ltd. (Jinan, China); and Dimethyl sulfoxide (DMSO) from Merck & Co., Inc. (Rahway, NJ, USA). Dulbecco’s modified Eagle medium (DEME) and Penicillin-Streptomycin were procured from Gibco Co., Ltd. (New York, NY, USA). Sodium chloride (NaCl) and anhydrous glucose were provided by Zhiyuan Chemistry Reagent Co., Ltd. (Tianjin, China). All the other chemicals and organic solvents were analytical reagents (ARs).

### 3.2. Preparation of 2-HP-β-CD/PLGA NPs

PLGA NPs were prepared according to the emulsification solvent evaporation method in Ref. [18] with minimal improvement. We dissolved 10 mg of the PLGA (LA/GA = 50:50, M_w_ 100,00 Da) carrier in 5 mL of the acetone/ethanol (4:1, *v*/*v*) solvent mixture to generate the oil phase; 1.5% 2-HP-β-CD-2% PVA aqueous solution (*m*/*v*, 50 mL) was the water phase. The oil phase was slowly injected into the water phase and emulsified for 10 min in an ice bath using a probe sonicator (JY88-IIN, Scientz Biotechnology Co., Ltd. (Ningbo, China) at 0–4 °C with an energy output of 150 W (varimax bar ψ6 mm) to develop an oil-in-water (O/W) emulsion. The PLGA NPs were procured by continuous low-speed stirring at room temperature until the organic solvent had evaporated entirely. Then, an appropriate amount of double-distilled water was fixed at 50 mL. 

The fluorescent labeling of the corresponding nanoparticles is required when studying the transport mechanism, thus facilitating the analysis of the intracellular localization characteristics of the nanoparticles. Rhodamine B (Rh-B) was selected as a fluorescent labeling dye to label the constructed nanoparticle nanosuspension in this experiment. In preparing the oil phase, we weighed appropriate amounts of Rh-B and PLGA (1:10, m/m) and dissolved them in a 50-times greater amount of the acetone/ethanol (4:1, *v*/*v*) solvent mixture. The Rh-B-(2-HP-β-CD)/PLGA NPs were prepared using the above method. In the experiments, the validated delivery system was evaluated in vivo for transport using triamcinolone acetonite (TA) as a model drug. TA-(2-HP-β-CD)/PLGA NPs were prepared in the same way as the Rh-B nanoparticle suspension, replacing Rh-B with an equal amount of TA to illustrate the efficiency of nanodelivery systems for enhancing corneal barrier permeation.

### 3.3. Characterization of 2-HP-β-CD/PLGA NPs

The particle size, polydispersity index (PDI), and zeta potential of the nanoparticles were essential factors affecting the corneal permeability and cellular uptake of the PLGA nanodelivery system [20]. Therefore, the experiments started by examining the nanometric properties of the synthesized PLGA nanoparticles.

The morphological observation of NPs was based on the literature on negative staining [28]. A few microliters of the sample solution was taken, dropped onto a copper grid covered with carbon film, and stained with 2% phosphotungstic acid solution for 10 min. After drying the grid, the morphology of the nanoparticles was observed using electron microscopy (TEM) (JEM-1400, Hitachi Ltd., Tokyo, Japan).

The particle size (diameter, nano); PDI; and zeta potential of Rh-B-(2-HP-β-CD)/PLGA NPs and TA-(2-HP-β-CD)/PLGA NPs were determined using a laser diffractometer (Delsa Nano CVR particle size and f-potential analyzer, Beckman Coulter Inc., CA, USA). The samples were diluted 10-fold using deionized water before the measurements at a controlled cell temperature of 25 ± 1 °C (*n* = 3).

### 3.4. Surface Tension

The surface tension of ophthalmic drops is essential in effectively adhering to and spreading in the cornea. Within a specific range, the lower the surface tension of an ophthalmic formulation, the more beneficial it is to mix the drops with the tear film, consequently improving the retention time of the cornea formulation. The surface tension of the nanoparticles was evaluated using the suspension drop method in an dense contact angle gauge (SDC-200, Sheng Ding Precision Instrument Co., Dongguan, China) optical contact angle measuring instrument.

### 3.5. Loading Capacity and Entrapment Efficiency

The entrapment efficiency (EE) and drug loading capacity (DL) of the nanoparticles were determined through a modified ultrafiltration technique [18]. The experiment began with low-speed centrifugation to separate the nanoparticles from the undissolved drug, with the undissolved drug settling at the bottom and the nanoparticles with a small quantity of dissolved drug on the upper liquid layer. Then, the upper layer was transferred to an ultrafiltration tube and centrifuged at high speed to separate the small amount of dissolved drug from the nanoparticles. HPLC was used to quantify the pharmaceutical content and estimate the EE and DL of the nanoparticle system based on the following equation:

$$EE(\%)=\frac{W_{1}-W_{2}}{W_{0}}\times 100\%$$

$$DL(\%)=\frac{(W_{1}-W_{2})V}{M}\times 100\%$$

where W_0_ = initial concentration of TA; W_1_ = concentration of TA in the upper liquid during low-speed centrifugation; W_2_ = concentration of TA in the ultrafiltrate; V = total volume of nanopreparations; M = total amount of (2-HP-β-CD)/PLGA NPs.

HPLC was performed using Waters HPLC equipment (e2695 HPLC system, Warters, MA, USA) and a Kromasil 100-5-C18 column (5 μm, 250 mm × 4.6 mm). The mobile phase was a binary water system: acetonitrile (58:42), with a flow rate of 1.0 mL·min^−1^ and the temperature maintained at 25 °C. The injection volume was 20 μL, and the detection wavelength was 240 nm. Later, a complete set of methodological validations was established, and the results satisfied the analytical assay requirements.

### 3.6. In Vitro Drug Release Study 

In vitro release characteristics represent a significant assessment of nanoparticle nanosuspension performance. In this paper, the dialysis bag method was used to demonstrate the release behavior of nanoparticles in vitro so that the structure and release mechanism of the nanoparticles could be illustrated. Precisely, 2 mL of TA aqueous suspension and TA-(2-HP-β-CD)/PLGA NPs was pipetted into a dialysis bag (molecular weight cut-off of 8 k-4 k), and the ends of the bag were taped tightly. Furthermore, the dialysis bags were immersed in centrifuge tubes containing 25 mL of release medium (artificial aqueous humor, pH 7.4) and shaken in a constant-temperature water bath (34 ± 0.5 °C, 100 rpm·min^−1^). Two milliliters of the release medium were withdrawn at 1, 2, 4, 6, 8, and 12 h, respectively, while the same fresh release medium was replenished. The formulations were filtered through a 0.22 μm microporous membrane and then subjected to HPLC, with similar chromatographic conditions maintained for the content determination to assess the cumulative percentage release. The equation for calculating the cumulative percentage of drug release is as follows:

$$Q=\frac{\left(C_{n}\times 25+V\sum_{i=1}^{n-1}C_{i}\right)}{C_{0}}\times 100$$

where C_n_ is the drug concentration at time t (μg·mL^−1^); C_i_ is the drug concentration at the last sampling time point t (μg·mL^−1^); C_0_ is the total amount of drug in the solution (μg·mL^−1^); and V is the volume of the sample obtained (2 mL).

### 3.7. Permeability Evaluation in Isolated Cornea 

Permeability is the dominant indicator of drug efficacy in entering the ocular tissue through the corneal epithelium. A modified Franz vertical diffusion cell helped us conduct the transcorneal permeability tests [29]. The TA aqueous suspension and TA-(2-HP-β-CD)/PLGA NPs were appraised using a rabbit cornea as a barrier. The pre-treated fresh isolated supracorneal layer was placed face-up and anchored between the supplies and receiving pools. The receptor compartment was filled with 5 mL of artificial aqueous humor (pH 7.4). In comparison, 0.5 mL of the sample (equivalent to 0.1 mg TA) was added to the epithelial side to obtain the donor compartment. The donor compartment was sealed with a sealing film, the device was kept at 34 ± 1 °C, and the stirring rate was maintained at 200 rpm. We obtained 2 mL of permeate medium from the receiving cell at 1, 2, 4, 6, and 8 h, respectively. The same volume of fresh release medium was also replenished.

HPLC was used to analyze the TA permeate, and all experiments were repeated three times. The chromatographic conditions for HPLC were the same as those used for content determination. The methodological validation was conducted using an in vitro drug release study, conforming to the linear range investigation, stability test, and precision test requirements.

Cumulative permeate volume (*Q*_n_, µg·cm^−2^); apparent permeability coefficient (*P*_app_, cm·s^−1^); and steady-state flow rate (*J*_ss_, µg·s^−1^·cm^−2^) were assayed using the following equations:

$$Q_{n}=\frac{V_{0}C_{n}+V\sum_{i-1}^{n-1}C_{i}}{A}$$

$$P_{app}=\frac{∆Q}{∆t\times C_{0}\times A\times 3600}$$

$$J_{ss}=C_{0}P_{app}$$

where V_0_ is the total volume of the medium inside the receptor compartment (5 mL); V is the volume of a single sample (2 mL); C_n_ is the drug concentration at time t (μg·mL^−1^); C_i_ is the drug concentration at the last sampling time point t (μg·mL^−1^); C_0_ is the initial drug concentration inside the supply pool (μg·mL^−1^); A is the effective transmission area of the Franz cell (0.5024 cm^2^); and ∆Q/∆t is the steady-state slope of the linear portion of the cumulative permeated drug quantity in the receptor compartment (Q) plotted versus time (t).

After the isolated cornea experiment, the remaining solution in the supply cell was discarded. The corneas were clipped at the exposed area of the diffusion cell, and the corneal surface was rinsed with saline to remove any nanoparticle residues. The corneas were chopped into a 10 mL centrifuge tube with 5 mL of methanol, vortexed, and sonicated for 15 min. Then, the samples were filtered through the 0.22 μm microporous membrane and injected, and HPLC was used to determine the corneal retention QR.

### 3.8. Pharmacokinetic Study of In Vivo Aqueous Humor

All animal protocols complied with the Guide for the Care and Use of Laboratory Animals and Institute of Laboratory Animal Resources and were approved by the Institutional Animal Care and Use Committee of Hainan Medical University and Guangdong Pharmaceutical University. Healthy New Zealand rabbits (weight 2–2.5 kg) were selected for the in vivo study. They were thoroughly checked before drug administration to exclude the effects of ocular disease. The microdialysis probe was implanted in the middle of the aqueous chamber according to a previous study [30]. When the probe was successfully implanted, one end was connected to the autosampler and set to collect a single sample for 30 min, and the other was linked to a syringe pump with a perfusion rate of 0.5 μL·min^−1^. Saline was used as the irrigation solution and perfused for 1 h to alleviate the minimally invasive local state. Subsequently, the underlying eyelids of the rabbits were lifted to create a pocket shape, through which 180 μL of the nanoformulation (1 drop at a time, six drops in total) was placed into the conjunctiva of the rabbit’s eyes, and the eyelids were closed briefly. The samplers were activated immediately to capture samples every 30 min and continued to operate for 6 h after dosing. The collections were stored at 15 °C without treatment and directly evaluated using HPLC (35 °C, injection volume of 5 μL; the rest of the conditions were the same as during content determination). The drug concentration in the atrial water (C_m_) was based on the equation below. In contrast, the pharmacokinetic analysis of the anterior chamber drug was conducted using the pharmacokinetic software DAS 3.0 to investigate the atrial chamber models of different formulations in terms of lg_C−t_.

$$C_{m}=\frac{C_{dialysis}}{R}$$

where C_dialysis_ is the drug concentration in aqueous humor, and R is the in vivo probe recovery rate.

### 3.9. Study of the Mechanism of Transport across the Cellular Barrier

After administering drops, the cornea is the primary barrier to drug delivery in the eye. In this study, human corneal epithelial cells (HCE-T) were used to construct an epithelial cell model for understanding the biological properties of the delivery system trans-keratocytes. Moreover, nanoparticle fluorescent probes loaded with Rh-B were used to investigate the transport mechanism across the epithelial cell barrier.

### 3.10. Cell Viability Assay

HCE-T cells (North Natronix Institute of Biotechnology, Beijing, China) were cultivated using a previously reported method [31]. The toxicity of the carrier material and the drug concentration are the foremost factors for investigating a drug delivery system. They indicate the mechanism of action and the strength of the delivery system, guiding the design of dosing regimens in future safety studies and clinical trials. The Cell Counting Kit-8 (CCK-8) approach was used to evaluate the cytotoxicity of nanoparticle fluorescent probes loaded with Rh-B. The following formula was implemented to analyze cell viability (%):

$$Cell\;viability(\%)=\frac{A_{s}-A_{b}}{A_{c}-A_{b}}\times 100\%$$

where A_s_ is the absorbance value for the experimental group, A_c_ is the absorbance value for the blank group, and A_b_ is the absorbance value for zeroing pores.

### 3.11. Study of the Uptake Process of Nanoparticles in HCE-T Cells

Nanoparticles were ingested by cells to determine their biological effects. The experiments were carried out by separately adding different cell functionality inhibitors. The impact of variations in cell-related functional properties leading to PLGA nanoparticle cell intake was analyzed.

### 3.12. Study of Active Transport Mechanism in Nanoparticle Uptake by HCE-T Cells

HCE-T cells were seeded at a density of 1 × 10^5^ cells·well^−1^ in a six-well plate, and the culture medium was discarded when the cell density reached 80%. First, 2 mL of 1 mg·mL^−1^ sodium azide was added and incubated for one hour. Then, fluorescently labeled nanoparticles were added for a final concentration of 20 μg·mL^−1^. After continuing the uptake experiment for 4 h, the fluorescently labeled nanoparticle solution was discarded, washed three times with PBS, and digested using trypsin. The cells were blown out with serum cultures, collected, and centrifuged at 1000 rpm. The supernatant was aspirated to remove the fluorescent material adsorbed on the cell surface. Finally, 0.3 mL of the cells suspended in PBS were transferred into flow tubes and assayed using flow cytometry (BD FACSCanto II, Becton, Dickinson and Company, New York, NY, USA). The fluorescence intensity was quantified by collecting 10,000 cells, and X-means were used for comparing the differences in cell uptake between the groups.

### 3.13. Study of Endocytic Transport Mechanism in Nanoparticle Uptake by HCE-T Cells

The main nanoparticle entry processes involve the clathrin structure, caveolin/lipid raft structure, and macropinocytosis [22]. The experiment was conducted by separately adding different endocytosis transport inhibitors to examine the degree of action during nanoparticle uptake. The clathrin structural inhibitor Chlorpromazine (30 μM), the caveolin/lipid raft structure inhibitor Nystain (30 μM), and the macropinocytosis inhibitor derivative amiloride (EIPA, 100 μM) were added seperately. Briefly, the cells were inoculated in six-well plates at a density of 1 × 10^5^ cells·well^−1^. The culture medium was discarded when the cell density reached approximately 80%. Serum-free cultures of varying endocytotic transport inhibitors were inspected using the method described above (Study of Active Transport Mechanism in Nanoparticle Uptake by HCE-T Cells).

### 3.14. Study of the Transport Mechanism of Nanoparticles in HCE-T Cells

Corneal epithelial cells are located on the outer overlying epithelial surface of the eye, which is characterized by cellular polarity. It is connected to the connective tissue through the basement membrane, behaving as a protective, secretory, and absorptive agent at the basal epithelial surface [23]. The monolayer cell barrier model constructed by HCE-T cells is a transmembrane structure used to study drugs and their corresponding agents [24]. Here, the qualitative and quantitative analysis of nanoparticle transport in HCE-T cells was analyzed with inverted fluorescence microscopy (IXM, Molecular Devices LLC, Sunnyvale, CA, USA) and flow cytometry (BD FACSCanto II, Becton, Dickinson and Company, New York, NY, USA).

### 3.15. Qualitative Analysis of Cellular Uptake of Nanoparticles

HCE-T cells were digested using trypsin/EDTA and inoculated in 24-well (1 mL·well^−1^) plates at a density of 1 × 10^5^ cells·well^−1^ after reaching 80% or more fusion. The culture solution was discarded; washed twice with PBS; and then added to serum-free culture solution with 20 μg·mL^−1^ fluorescently labeled nanoparticles and cultured for 0.5, 1, 2, and 4 h after taking the samples.

Finally, the nanoparticles were discarded and washed thrice with PBS. Afterward, the cells were fixed at room temperature for 15 min by adding 4% paraformaldehyde solution (dissolved in PBS). Then, they were discarded and washed thrice with PBS. IXM was used to observe the uptake of fluorescently labeled nanoparticles by HCE-T cells.

### 3.16. Quantitative Analysis of Cellular Uptake of Nanoparticles

HCE-T cells were extracted using trypsin/EDTA and inoculated in six-well plates at a density of 1 × 10^5^ cells·well^−1^. When the cell density reached 80%, the culture medium was discarded and washed twice using PBS. Then, the culture was maintained at 0.5, 1, 2, and 4 h in a serum-free medium with 20 μg·mL^−1^ fluorescently labeled nanoparticles. The nanoparticle solution was discarded after the experiment. The cells were treated according to the method described above (Study of Active Transport Mechanism in Nanoparticle Uptake by HCE-T Cells).

### 3.17. Data Analysis and Statistics

All the data in this study are expressed as mean ± SD and were statistically analyzed using GraphPad Prism 9.0 and Origin 64Bit. One-way analysis of variance (ANOVA) methodology was applied to ascertain the significance of the pilot data. Pharmacokinetic data manipulation was undertaken using pharmacometrics software (DAS 3.0). *p*-values less than 0.01 were considered to be significantly different, *p*-values less than 0.05 were regarded as statistically different, and *p*-values greater than 0.05 were deemed not significantly different.

## 4. Conclusions

In this study, TA-PLGA NPs modified with 2-HP-β-CD were prepared and validated through cellular experiments, showing that TA-(2-HP-β-CD)/PLGA NPs could disrupt the membrane structure. The drug could effectively penetrate biological membranes with an altered entry mode for the nanoparticles, effectively enhancing the corneal permeability of the drug. In vitro studies demonstrated that this drug delivery system exhibited a sustained release, prolonging the anterior corneal retention time and developing reservoirs in the corneal epithelium, thereby sustaining the driving force for drug diffusion into the aqueous humor and effectively enhancing the quantity of drug penetration through the cornea. Aqueous kinetic tests revealed that the nanosystem was less susceptible to tear flushing, could effectively transcend the corneal barrier, maintained the drug concentration in the aqueous humor, and provided high bioavailability. This study provides essential information on (2-HP-β-CD)/PLGA nanocarriers as a novel non-invasive drug delivery technology and presents innovative ideas for developing transocular barrier nanopreparations.

## Figures and Tables

**Table 1:** The particle size and zeta potential of (2-HP-β-CD)/PLGA NPs (mean ± SD, *n* = 3).

	Particle Size (nm)	PDI	Zeta (mV)
Rh-B-(2-HP-β-CD)/PLGA NPs	182.73 ± 0.32	0.062 ± 0.018	−27.19 ± 4.20
TA-(2-HP-β-CD)/PLGA NPs	132.70 ± 3.34	0.102 ± 0.017	−13.63 ± 2.65

**Table 2:** The release models of triamcinolone acetonide in vitro.

	Release Model	Fitting Equation	*R* ^2^
TA aqueous suspension	Zero-order	Q = 1.8758t + 83.323	0.4571
	First-order kinetics	ln(100-Q) = −0.5616t + 3.0702	0.9224
	Higuchi	Q = 9.6624t^1/2^ + 72.453	0.6031
	Ritger–Peppas	lnQ = 0.1245lnt + 4.3574	0.7367
TA-(2-HP-β-CD)/PLGA NPs	Zero-order	Q = 6.8146t + 31.195	0.6797
	First-order kinetics	ln(100-Q) = −0.2735t + 4.4202	0.9318
	Higuchi	Q = 33.554t^1/2^ − 4.9001	0.8195
	Ritger–Peppas	lnQ = 0.8687lnt + 2.7863	0.7947

**Table 3:** *P*_app_, *J*_ss_, and *Q*_R_ of the nanoparticles in the excised corneal penetration test (mean ± SD, *n* = 3).

Parameter	*P*_app_ (×10^−7^ cm·s^−1^)	*J*_ss_ (×10^−3^ μg·s·cm^−2^)	*Q*_R_ (μg·cm^−2^)
TA aqueous suspension	8.35 ± 1.02	0.16 ± 0.02	2.19 ± 0.19
TA-(2-HP-β-CD)/PLGA NPs	68.85 ± 7.73	1.33 ± 0.20	16.06 ± 3.96

**Table 4:** The drug concentration effect on microdialysis probe recoveries in vitro (mean ± SD, *n* = 3).

Formulation	*C*_max_	${AUC}_{0\to t}$	MRT	*t*_max_	VRT
	mg·L^−1^	h·h·mg·L^−1^	h	h	h^2^
TA aqueous suspension	0.297 ± 0.056	0.481 ± 0.059	2.048 ± 0.089	1.5 ± 0	0.711 ± 0.048
TA-(2-HP-β-CD)/PLGA NPs	15.418 ± 2.635	25.267 ± 0.744	2.116 ± 0.214	1.5 ± 0	1.477 ± 0.669

## Data Availability

The original contributions presented in the study are included in the article; further inquiries can be directed to the corresponding authors.

## Abbreviations

Abbreviation	Complete Name
TA	Triamcinolone acetonide
Rh-B	Rhodamine B
PVA	Polyvinyl alcohol
2-HP-β-CD	2-Hydroxypropyl-β-cyclodextrin
PLGA	Poly(lactic acid glycolic acid) copolymer
PBS	Phosphate-buffered saline
DMSO	Dimethyl sulfoxide
DEME	Dulbecco’s modified Eagle medium
